# Continued occurrence of serotype 1 pneumococcal meningitis in two regions located in the meningitis belt in Ghana five years after introduction of 13-valent pneumococcal conjugate vaccine

**DOI:** 10.1371/journal.pone.0203205

**Published:** 2018-09-07

**Authors:** Catherine H. Bozio, Abass Abdul-Karim, John Abenyeri, Braimah Abubakari, Winfred Ofosu, Justina Zoya, Mahamoudou Ouattara, Velusamy Srinivasan, Jeni T. Vuong, David Opare, Franklin Asiedu-Bekoe, Fernanda C. Lessa

**Affiliations:** 1 Division of Bacterial Diseases, National Center for Immunization and Respiratory Diseases, Centers for Disease Control and Prevention, Atlanta, GA, United States of America; 2 Epidemic Intelligence Service, Centers for Disease Control and Prevention, Atlanta, GA, United States of America; 3 Zonal Public Health Laboratory, Tamale, Ghana; 4 Northern Regional Directorate, Tamale, Ghana; 5 Upper West Regional Directorate, Wa, Ghana; 6 National Public Health Reference Laboratory, Ghana Health Service, Accra, Ghana; 7 Ghana Health Service, Accra, Ghana; Universidade de Lisboa Faculdade de Medicina, PORTUGAL

## Abstract

**Background:**

Increases in pneumococcal meningitis were reported from Ghanaian regions that lie in the meningitis belt in 2016–2017, despite introduction of 13-valent pneumococcal conjugate vaccine (PCV13) in 2012 using a 3-dose schedule (6, 10, and 14 weeks). We describe pneumococcal meningitis epidemiology in the Ghanaian Northern and Upper West regions across two meningitis seasons.

**Methods:**

Suspected meningitis cases were identified using World Health Organization standard definitions. Pneumococcal meningitis was confirmed if pneumococcus was the sole pathogen detected by polymerase chain reaction, culture, or latex agglutination in cerebrospinal fluid collected from a person with suspected meningitis during December 2015-March 2017. Pneumococcal serotyping was done using PCR. Annual age-specific pneumococcal meningitis incidence (cases per 100,000 population) was calculated, adjusting for suspected meningitis cases lacking confirmatory testing.

**Findings:**

Among 153 pneumococcal meningitis cases, 137 (89.5%) were serotyped; 100 (73.0%) were PCV13-type, including 85 (62.0%) that were serotype 1, a PCV13-targeted serotype. Persons aged ≥5 years accounted for 96.7% (148/153) of cases. Comparing 2015–2016 and 2016–2017 seasons, the proportion of non-serotype 1 PCV13-type cases decreased from 20.0% (9/45) to 4.1% (3/74) (p = 0.008), whereas the proportion that was serotype 1 was stable (71.1% (32/45) vs. 58.1% (43/74); p = 0.16). Estimated adjusted pneumococcal meningitis incidence was 1.8 in children aged <5 years and ranged from 6.8–10.5 in older children and adults.

**Conclusions:**

High pneumococcal meningitis incidence with a large proportion of serotype 1 disease in older children and adults suggests infant PCV13 vaccination has not induced herd protection with this schedule in this high-transmission setting.

## Introduction

Seasonal increases in pneumococcal meningitis have been reported in the African meningitis belt prior to pneumococcal conjugate vaccine (PCV) introduction, with case-fatality ratios as high as 50% in some age groups [1]. The African meningitis belt spans from Senegal to Ethiopia and experiences annual seasonal meningitis outbreaks with large-scale epidemics every 8–12 years [2]. These outbreaks typically occur during the dry season, as humidity, dry harmattan wind, and dusty conditions increase the risk of bacterial meningitis [3]. Ghana has three regions (Northern, Upper West, and Upper East regions) in the meningitis belt. Between 2002 and 2003 (pre-PCV introduction), Ghana experienced epidemics of pneumococcal meningitis similar to those in Burkina Faso, which borders the Upper East and Upper West Regions of Ghana. In both of these countries, the outbreaks were caused by serotype 1 pneumococci, occurred early in the meningitis season, and affected mainly children aged <5 years [4, 5]. With the support of Gavi, the Vaccine Alliance, Ghana introduced 13-valent PCV (PCV13; serotypes 1, 3, 4, 5, 6A, 6B, 7F, 9V, 14, 18C, 19A, 19F, and 23F) in their national infant immunization program in 2012 using a three-dose primary series schedule (3+0) at 6, 10, and 14 weeks of age without a booster or catch-up campaign. In 2013–2016, administrative coverage estimates for three PCV13 doses were 88–93% [6].

In the 2015–2016 meningitis season, a large outbreak of serotype 1 pneumococcal meningitis occurred in the Brong-Ahafo region in Ghana, which falls outside of the meningitis belt. Nearly 60% of pneumococcal meningitis cases occurred in persons aged 5–29 years, with children aged <5 years accounting for 4.7% of cases [7]. In contrast, the Northern, Upper West, and Upper East regions experienced an increase in pneumococcal meningitis early in the season [8]. In February 2017, districts in the Upper West and Northern regions reported an increased number of suspected meningitis cases, with four districts (two in each region) crossing the World Health Organization (WHO) established epidemic threshold of 10 cases per 100,000 population [9]. Most of these initial cases were confirmed to be due to pneumococcus. We describe the epidemiology of pneumococcal meningitis across two meningitis seasons (2015–2016, 2016–2017) in the Upper West and Northern regions of Ghana, five years after PCV13 introduction into the routine infant immunization program.

## Methods

### Data collection

Case-based meningitis surveillance has been ongoing in the three northern regions of Ghana since 2013. Detailed epidemiological data (including outcome status) are collected using a standardized case report form for each patient who meets the WHO standard definition of suspected meningitis [10]. Cerebrospinal fluid (CSF) specimens collected from patients with suspected meningitis are sent to district- or regional-level laboratories, along with a case report form, for bacteriologic testing, which includes latex agglutination, Gram stain, and, where available, culture. Laboratory test results are entered on the case report form. CSF specimens and case report forms are transmitted to the Tamale Zonal Public Health laboratory (hereafter referred to as Tamale laboratory), the reference laboratory for meningitis in Ghana, where direct real-time polymerase chain reaction (PCR) for detection and serotyping/serogrouping of vaccine-preventable bacterial pathogens are performed. Laboratory data are compiled into a laboratory line list that can be merged with the surveillance data using a unique epidemiologic number that is assigned to each suspected case.

We used case-based surveillance data to identify suspected meningitis cases with dates of symptom onset from December 1, 2015 through March 31, 2017. We then compiled case-based surveillance data with Tamale laboratory data. Using WHO guidelines [10], a suspected case of bacterial meningitis was defined as sudden onset of fever (>38.5°C rectal or >38.0°C axillary) plus neck stiffness, bulging fontanelle, convulsions, altered consciousness or other meningeal signs, in a resident of any district located in the Upper West or Northern regions. A confirmed case of bacterial meningitis was defined as a suspected case with a bacterial pathogen detected by PCR, culture, or latex agglutination. However, a pneumococcal meningitis case was considered to be confirmed only if pneumococcus was the single pathogen detected.

The meningitis surveillance is considered a public health activity and was granted a non-research by the U.S. Centers for Disease Control and Prevention (CDC) Institutional Review Board. The surveillance protocol was approved by Ghana Health Services Ethical Review Committee as research with minimal risk and informed consent was waived (GHS-ERC 16.07.2016). No additional data beyond what is part of routine surveillance was obtained. A unique identifier was assigned for each patient by the Regional Public Health Surveillance office.

### Laboratory methods

Frozen CSF specimens from local hospitals were transported to the Tamale laboratory on ice packs and stored at -20°C until testing. No isolates were sent to the Tamale laboratory. PCR assays were used for detection of *Streptococcus pneumoniae*, *Neisseria meningitidis*, and *Haemophilus influenzae*, using gene targets of *lytA*, *sodC*, and *hpd*, respectively [11]. A cycle threshold (Ct) value ≤36 was considered positive for each bacterium.

Serotyping and/or serogrouping was done only on CSF specimens positive for any of the pathogens tested by PCR. *S*. *pneumoniae* serotyping was done directly from *S*. *pneumoniae-*positive CSF specimens, using seven sequential triplex PCR reactions to detect 37 serotypes [12]. Briefly, the surface of the cryovials containing CSF specimen was carefully disinfected with paper towel treated in 10% of commercial bleach and vortexed for 5–10 seconds to homogenize the CSF. Two μl of the homogenized CSF specimen were added into PCR tube containing 23μl of master mix, 2X Quanta Biosciences PerfeCTa qPCR ToughMix (Thermo Fisher Inc.) and double the original concentration of primers and probes described in Pimenta et al. [12]. PCR was performed and results were analyzed on AriaMx instrument and software (Agilent Technologies, Santa Clara, USA).

### Statistical methods

Pneumococcal meningitis was defined as PCV13-type if any of the following *S*. *pneumoniae* serotypes were identified in a *S*. *pneumoniae*-positive CSF specimen: 1, 3, 4, 5, 6A, 6B, 7F, 9V/9A, 14, 18C/18F/18B/18A, 19A, 19F, or 23F; all other pneumococcal meningitis was defined as non-PCV13 type. Age groups were categorized as <5, 5–14, 15–29, 30–59, and ≥60 years. Annual overall and age-specific pneumococcal meningitis incidence rates (cases per 100,000 population) were calculated based on data from epidemiologic week 14 in 2016 through epidemiologic week 13 in 2017, using the population estimated from the twenty districts where pneumococcal meningitis patients resided. To adjust for lack of confirmatory testing in some patients, the total number of confirmed pneumococcal meningitis cases in each age and region stratum was divided by the number of suspected cases in that stratum with CSF tested at the Tamale laboratory; this proportion was then applied to cases without CSF samples sent to the Tamale laboratory within that age and region stratum (hereafter referred to as “imputed” results). Two numerators were used for rate calculations: 1) *S*. *pneumoniae* detected by PCR, culture, or latex agglutination, and 2) Observed plus imputed *S*. *pneumoniae* meningitis cases. Cases were summed across region by age stratum to obtain age-specific incidence rates. Proportions were compared across demographic variables using Pearson’s chi-square or Fisher’s exact test. When comparing proportions of PCV13-type pneumococcal meningitis including and excluding serotype 1 between two meningitis seasons, Bonferroni’s correction was applied to adjust the p-value for multiple comparisons. A p-value less than 0.05 was considered statistically significant; the Bonferroni-corrected p-value threshold was 0.0125. All analyses were conducted using SAS 9·4 (Cary, NC).

## Results

From December 1, 2015 through March 31, 2017, 1,614 suspected meningitis cases were reported in the Upper West and Northern regions, of which 1,552 (96.2%) had CSF specimens collected (Table 1). The proportion of suspected meningitis cases with CSF collected did not significantly differ by age or region (both p>0.05). Of 1,552 CSF specimens collected, 796 (51.3%) were sent to the Tamale laboratory for further analysis. The proportions of CSF specimens received at the Tamale laboratory were significantly lower in children aged <5 years (40.4%) and adults aged ≥60 years (42.1%), compared to the other age groups (p<0.0001). Tamale laboratory received significantly more CSF specimens from the Northern Region, where the laboratory is located, compared to the Upper West region (70.2% vs. 41.8%; p<0.0001).

**Table 1:** Characteristics of suspected meningitis cases with symptom onset from December 1, 2015 through March 31, 2017.

Characteristic		Number of suspected meningitis cases	Number of cases with CSF collected	Proportion of suspected cases with CSF collected	Number of CSF specimens tested by Tamale laboratory	Proportion of cases with CSF collected that were tested by Tamale laboratory	Confirmed*	*S*. *pneumoniae*	*N*. *meningitidis*	*H*. *influenzae*	>1 pathogen detected†
Age (years)	<5	280	275	98.2%	111	40.4%	48	4	40	1	3
	5–14	424	410	96.7%	232	56.6%	151	47	101	1	2
	15–29	450	432	96.0%	234	54.2%	113	48	59	3	3
	30–59	312	294	94.2%	165	56.1%	72	46	22	0	4
	≥60	140	133	95.0%	56	42.1%	20	7	10	1	2
	Missing	8	8	100.0%	3	37.5%	5	1	4	0	0
Region	Upper West	1020	985	96.6%	412	41.8%	168	81	77	4	6
	Northern	594	567	95.5%	398	70.2%	241	72	159	2	8
Sex	Male	833	808	97.0%	412	51.0%	215	80	122	3	10
	Female	780	743	95.3%	383	51.5%	189	71	112	3	3
	Missing	1	1	100.0%	1	100.0%	5	2	2	0	1
Total		1614	1552	96.1%	796	51.3%	409	153	236	6	14

*Confirmed by direct real-time polymerase chain reaction (PCR), culture, or latex agglutination as *Streptococcus pneumoniae*, *Neisseria meningitidis*, and/or *Haemophilus influenzae*.

†Of the 14 specimens that had more than 1 pathogen detected, 10 had *N*. *meningitidis* and *S*. *pneumoniae* detected, and 4 had *S*. *pneumoniae* and *H*. *influenzae* detected.

Among the 153 confirmed pneumococcal meningitis cases, 148 (96.7%) occurred in persons aged ≥5 years. Of the 98 pneumococcal meningitis cases with a known outcome status, 28 (28.6%) patients died. The case-fatality ratio for pneumococcal meningitis was similar in older children and adults: 0% (0/2) in children aged <5 years, 27.6% (8/29) in ages 5–14 years, 25.0% (8/32) in ages 15–29 years, 25.8% (8/31) in ages 30–59 years, and 100% (4/4) in ages ≥60 years.

Of the 153 pneumococcal meningitis cases, 92 were positive by PCR, six by culture, three by latex agglutination, and 52 by multiple methods; 16 (10.5%) did not have a CSF specimen available at Tamale laboratory for serotyping. Of the 137 pneumococcal meningitis cases serotyped, 100 (73.0%) were PCV13-type, 35 (25.5%) were non-PCV13 type, and two (1.5%) had multiple serotypes detected and were considered inconclusive. Of the 100 PCV13-type pneumococcal meningitis cases, 85 (85.0%) were serotype 1; followed by serotype 5 (4.0%), serotype 23F (3.0%), serotype 18C/18F/18B/18A (2.0%), serotype 3 (1.0%), serotype 4 (1.0%), serotype 6A/6B (1.0%), serotype 14 (1.0%), serotype 19A (1.0%), and serotype 19F (1.0%). Of the 35 non-PCV13 serotypes, 19 (54.3%) were serotype 12F/12A/12B/44/46 and 16 (45.7%) were negative for the serotypes included in the PCR reaction. Of the 85 serotype 1 pneumococcal meningitis cases, one (1.2%) occurred in persons aged <5 years, 33 (38.8%) in persons aged 5–14 years, 28 (32.9%) in persons aged 15–29 years, 19 (22.4%) in persons aged 30–59 years, and three (3.5%) in persons aged ≥60 years; age was not reported for one person. Among the four confirmed pneumococcal meningitis cases in children aged <5 years, two were PCV13-type: one was serotype 1 and one was 19F; both had unknown PCV13 vaccination status.

**Fig 1:**
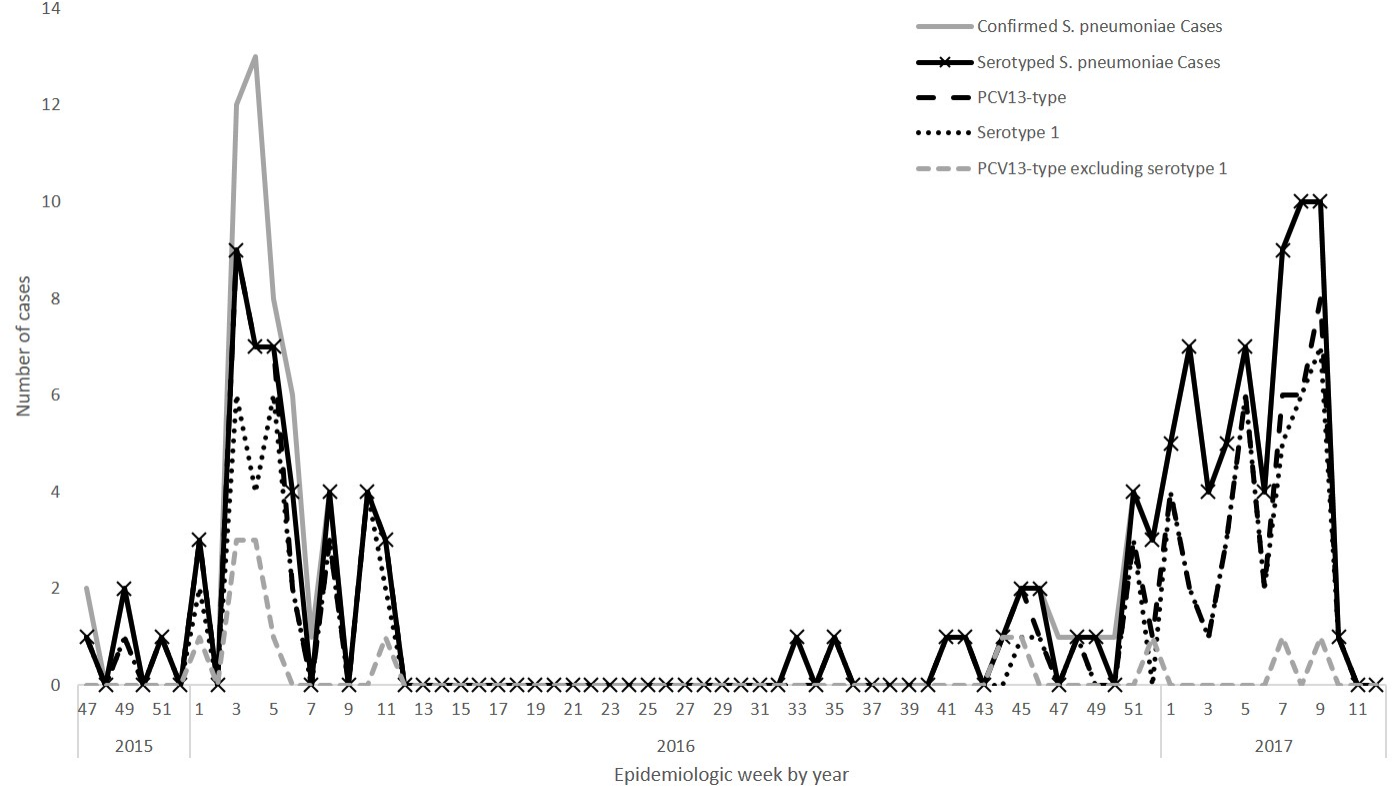
Epidemiologic curve of confirmed* pneumococcal meningitis cases, serotyped cases, PCV13 serotypes, and serotype 1 by epidemiologic week between December 1, 2015 and March 31, 2017. *S. *pneumoniae* detected in cerebrospinal fluid (CSF) by direct real-time polymerase chain reaction or latex or isolated from CSF by culture.

During the first meningitis season (December 1, 2015-June 30, 2016 [epidemiologic weeks 48–26 in 2015–2016]), 897 suspected meningitis cases were reported; 373 CSF specimens were sent to the Tamale laboratory, of which 59 had pneumococcus detected. Thirty-seven of these 59 pneumococcal meningitis cases were included in a previous publication describing the 2016 meningitis outbreak in Ghana [8]. In comparison, during the second meningitis season (December 1, 2016-March 31, 2017 [epidemiologic weeks 48–13 in 2016–2017]), 473 suspected meningitis cases were reported; 354 CSF specimens were sent to the Tamale laboratory, of which 75 had pneumococcus detected (Fig 1). The proportion of PCV13 serotypes among serotyped pneumococcal meningitis cases significantly decreased from 91.1% (41/45) to 62.2% (46/74) (p = 0.0004). The proportion that were non-serotype 1 PCV13-type decreased from 20.0% (9/45) to 4.1% (3/74) (p = 0.008) between the two meningitis seasons, while the proportion that were serotype 1 was stable (71.1% (32/45) in season 1 vs. 58.1% (43/74) in season 2 (p = 0.16)). However, the proportion of serotyped pneumococcal meningitis due to 12F/12A/12B/44/46, a non-PCV13 serotype, increased from 4.4% (2/45) in the first season to 18.9% (14/74) in the second season; however, this increase was not significant after Bonferroni correction.

**Fig 2:**
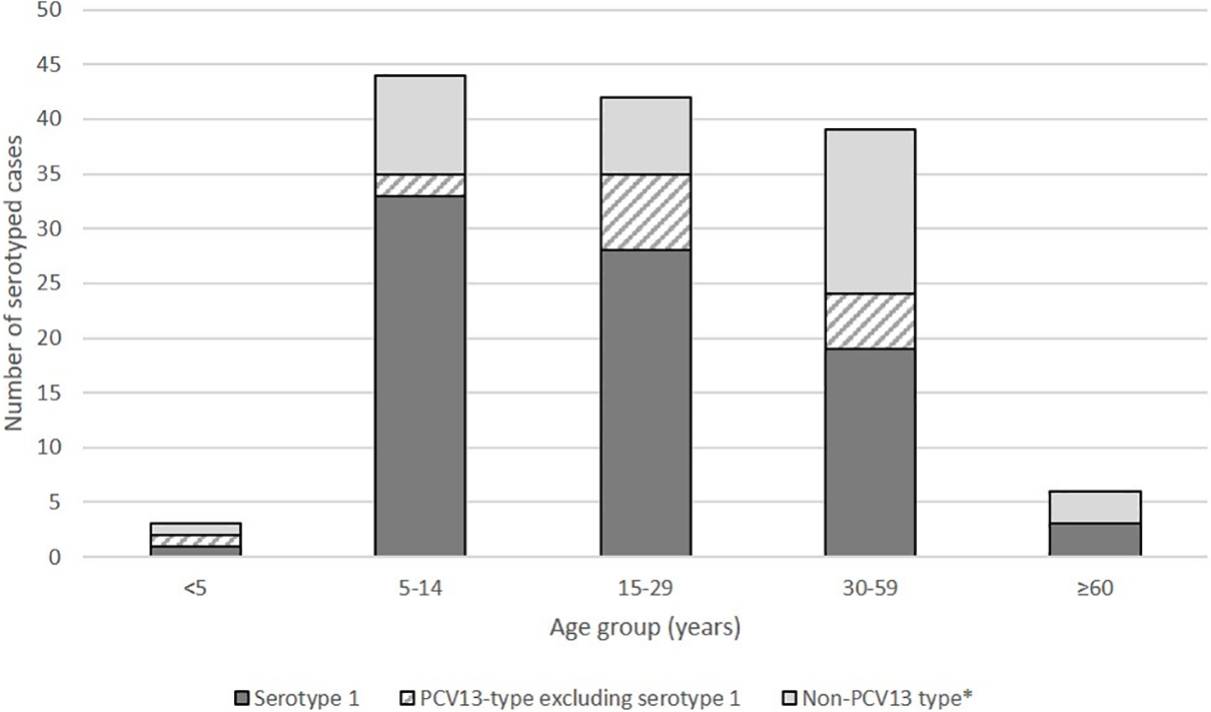
Serotype distribution of pneumococcal meningitis cases by age group, December 1, 2015-March 31, 2017. *Includes pneumococcal samples negative for the 37 serotypes tested by PCR.

Among serotyped pneumococcal cases in persons aged ≥5 years, 97 (74.0%) were PCV13-type, including 80 (64.0%) serotype 1. Further, the proportion that were serotype 1 ranged from 48.7% in persons aged 30–59 years to 75.0% in persons aged 5–14 years, but the overall pneumococcal serotype distribution was not statistically different across age groups (p = 0.058) (Fig 2).

From epidemiologic weeks 14–13 in 2016–2017, 85 confirmed pneumococcal meningitis cases were reported from 20 districts for an annual observed incidence of 4.33/100,000 (Table 2). Children aged <5 years had the lowest observed pneumococcal meningitis incidence (0.64 cases per 100,000), compared to other age groups. After adjusting for suspected meningitis cases lacking confirmatory testing, the imputed incidence increased by 1.4–2.8 times. These increases were highest among persons aged <5 and ≥60 years (2.8 and 2.3 times higher, respectively), as they had the lowest proportions of cases with CSF collected for which specimens were received at the Tamale laboratory. The imputed incidence was highest in adults aged ≥60 years and 30–59 years (10.45 and 10.51 per 100,000 population, respectively) (Table 2).

**Table 2:** Incidence of confirmed pneumococcal meningitis cases by age group for epidemiologic weeks 14 in 2016 through 13 in 2017.

				Incidence rate (cases per 100,000 population)	
Age (years)	Observed	Imputed	Population*	Observed	Imputed
<5	2	5.56	310,349.01	0.64	1.79
5–14	30	43.15	523,713.95	5.73	8.24
15–29	21	35.69	523,713.95	4.01	6.81
30–59	26	47.49	451,945.74	5.75	10.51
≥60	6	13.58	129,958.65	4.62	10.45
Total	85	145.47	1,939,681.29	4.33	7.50

*This population is the sum of the 20 districts from which the 84 confirmed pneumococcal meningitis resided.

## Discussion

Despite Ghana’s introduction of PCV13 in 2012 and subsequent high vaccination coverage, vaccine-type pneumococci continues to be a main cause of bacterial meningitis in the Northern and Upper West regions. Although children aged <5 years had the lowest burden of pneumococcal meningitis, most pneumococcal meningitis cases and serotype 1 cases occurred in persons aged ≥5 years, who were not age-eligible to have received PCV13. Additionally, across the two meningitis seasons, the proportion of pneumococcal meningitis cases caused by serotype 1 did not change although the proportion caused by other PCV13-types declined. These findings suggest that, while PCV13 introduction produced the anticipated direct effect in Ghana, herd immunity, at least for serotype 1, has not yet been established.

From pre-PCV introduction data in West African countries, including Ghana [1, 4, 5, 13], pneumococcal meningitis affected all ages, with highest rates in children aged <5 years. From 2015–2017, Ghanaian children aged <5 years had dramatically lower rates compared to those pre-PCV introduction, suggesting PCV13’s direct effect. In Ghana’s neighboring country, Burkina Faso, where PCV13 was introduced in 2013 using a 3+0 schedule, children aged <1 and 1–4 years had the largest decreases in rates for confirmed pneumococcal meningitis, PCV13-type pneumococcal meningitis, and serotype 1 pneumococcal meningitis by 2015 [14]. In Mozambique, following PCV10 introduction using a 3+0 schedule, the proportion of confirmed bacterial meningitis cases that were pneumococcal decreased from 33.6% in 2013 to 1.9% in 2015 among children aged <5 years [15]. Finally, in South Africa, the incidence of serotype 1 invasive pneumococcal disease (IPD) (cases per 100,000 population) decreased significantly in children aged <1 year (2003–2008: 1.8 cases vs 2013: 0.3 cases) and 1–4 years (2003–2008: 1.3 cases vs 2013: 0.3 cases), after PCV13 introduction using a schedule of two primary doses and one booster dose (2+1) [16].

Despite high PCV13 vaccine coverage in young children, the burden of PCV13-type pneumococcal meningitis, especially serotype 1, remains high in older children and adults in Ghana, suggesting that herd effects for serotype 1 have not yet been established. A higher prevalence of risk factors for other bacterial diseases spread through droplets among adults may contribute to this shift in the age distribution of disease burden [17]. During the 2015–2016 pneumococcal meningitis outbreak in Ghana’s Brong-Ahafo region, 94.1% of confirmed pneumococcal meningitis cases were aged ≥5 years; among persons aged ≥5 years, 71.4% had serotype 1 disease [7]. In Burkina Faso, serotype 1 pneumococcal meningitis incidence in children aged 5–14 years also remained stable after PCV13 introduction using a 3+0 schedule [14]. In contrast, in South Africa, which adopted a 2+1 schedule, serotype 1 IPD incidence decreased significantly by 2013 across all age groups except persons aged >64 years [16].

The lack of indirect effect of PCV for serotype 1 using a 3+0 schedule observed in countries in the meningitis belt raises concerns. A trial of PCV9 (which includes serotype 1) efficacy in the Gambia showed that PCV9 was not effective against serotype 1 disease, although this was based on only six isolates [18]. In a randomized trial comparing four PCV13 schedules, the immune response (one month after the primary series) against serotype 1 with the 2-3-4 month schedule (which is most similar to Ghana’s and Burkina Faso’s schedules) was inferior compared to the 2-4-6 month, 3–5 month, and 2–4 month schedules [19]. Waning immunity for serotype 1 is a concern for PCV13 schedules without a booster dose [20, 21]. In the 12 IPD cases of serotype 1 vaccine failures identified from PCV9 trials in Africa, the median age was 20.9 months (range 18.4–27.5 months), which may reflect failures in the absence of a booster dose [21]. Additionally, in a PCV10 trial, opsonophagocytic activity against serotype 1 was lower relative to other PCV-types one month after the primary series [22], which can impact both carriage and disease. Thus, a booster dose after the primary series may be needed to provide sufficient protection against serotype 1 disease.

The relative decline of non-serotype 1 PCV13-types among serotyped pneumococcal meningitis cases was followed by an increase in serotype 12F/12A/12B/44/46. Serotype 12F was the second most common serotype in the Brong-Ahafo outbreak in 2016 [7] and is associated with antimicrobial resistance [23], leading to concerns that this serotype might emerge as a source of replacement disease post-PCV introduction.

Our analysis has limitations. First, not all CSF specimens collected were sent to Tamale for PCR testing. In particular, a larger proportion of CSF specimens collected from the Northern region compared to the Upper West region were received at the Tamale laboratory, although we accounted for this difference in imputing pneumococcal meningitis rates for suspected meningitis cases without confirmatory testing. Thus, it is possible that we underestimated the number of bacterial meningitis cases. Additionally, 13 (8.4%) patients with pneumococcal meningitis had another pathogen detected. These cases were conservatively excluded from the analyses due to concerns of potential cross-contamination given that meningitis caused by more than one bacterial pathogen is rare. Therefore, it is possible that the number of pneumococcal meningitis cases was underestimated.

Despite high PCV13 vaccination coverage, Ghana continues to experience a substantial burden of pneumococcal meningitis, primarily affecting older children and adults, in the Northern and Upper West regions. It is unclear whether PCV vaccination in infancy prevents pneumococcal meningitis outbreaks, and concerns about waning immunity for serotype 1 in PCV13-vaccinated persons in countries that adopted a 3+0 schedule have been raised [24]. Alternative vaccination strategies could be explored to protect persons of all ages from pneumococcal meningitis, especially that due to serotype 1. Ongoing work to identify the drivers of carriage and transmission, and continued monitoring of the epidemiology of pneumococcal meningitis could help to inform development and application of these strategies, particularly in the African meningitis belt region, where meningitis incidence is high and epidemics occur.

## Supporting information

S1 Dataset (XLSX)
